# Influence of a Municipal Waste Landfill on the Spatial Distribution of Mercury in the Environment

**DOI:** 10.1371/journal.pone.0133130

**Published:** 2015-07-15

**Authors:** Barbara Gworek, Wojciech Dmuchowski, Dariusz Gozdowski, Eugeniusz Koda, Renata Osiecka, Jan Borzyszkowski

**Affiliations:** 1 Institute of Environmental Protection–National Research Institute, Warsaw, Poland; 2 Warsaw University of Life Sciences–SGGW, Warsaw, Poland; 3 Botanical Garden–Centre for Biological Diversity Conservation, Polish Academy of Sciences, Warsaw, Poland; CSIR- Indian Institute of Toxicology Research, INDIA

## Abstract

The study investigations were focused on assessing the influence of a 35-year-old municipal waste landfill on environmental mercury pollution. The total Hg content was determined in the soil profile, groundwater, and the plants (*Solidago virgaurea* and *Poaceae* sp.) in the landfill area. Environmental pollution near the landfill was relatively low. The topsoil layer, groundwater and the leaves of *Solidago virgaurea* and *Poaceae* sp. contained 19–271 μg kg^-1^, 0.36–3.01 μg l^-1^, 19–66 μg kg^-1 ^and 8–29 μg kg^-1^ of Hg, respectively. The total Hg content in the soil decreased with the depth. The results are presented as pollution maps of the landfill area based on the total Hg content in the soil, groundwater and plants. Statistical analysis revealed the lack of correlation between the total Hg content in the soil and plants, but a relationship between the total concentration of Hg in groundwater and soil was shown. The landfill is not a direct source of pollution in the area. The type of land morphology did not influence the pollution level. Construction of bentonite cut-off wall bypassing MSW landfill reduces the risk of mercury release into ground-water environment.

## Introduction

Mercury is a global contaminant posing severe risks to the health of ecosystems and humans worldwide. The environmental contamination of land, air, water, and wildlife in various ecosystems with Hg around the world due to the natural release and extensive anthropogenic use of Hg has been a global concern for decades [1].

Global emissions of Hg in 2005 from landfills and waste utilization are estimated at 187 Mg, which is 8.1% of the total emissions from anthropogenic sources. However, the estimate from this sector exhibits large uncertainties due to the lack of field measurement data [2]. India produces the most Hg, with a measurement of 77.4 Mg [3], followed by China with 14.1 Mg [4], North America with 13.0 Mg [5] and Europe with 10.1 Mg [6].

Landfilling remains the predominant management method for the disposal of municipal solid waste (MSW) in Poland. Used batteries, electric equipment, lighting equipment, control-measuring devices, mercury amalgams and used paint tins are common items in municipal waste landfills [7]. Wastes containing Hg that are deposited in landfills may become long-term sources of environmental pollution to the air, water and land through leaching [8].

The Hg content in municipal wastes is difficult to determine. Hg is occasionally recovered from waste, but this is often financially inviable. Concentration of Hg in municipal landfill can range from 0.033 to 46.2 mg kg^-1^ [9,10]. Intense source reduction efforts have been implemented to reduce the Hg content in municipal waste landfills, resulting in a rapidly declining trend from 1.8 mg Hg/kg in 1995 to 0.5 mg Hg/kg in 2009 [11]. However, the increasing quantities of MSW generated by society constantly adds to increasing the Hg load into landfills.

Landfill leachate contains a variety of pollutants that may potentially contaminate the groundwater and affect the quality of surface and well waters. Concentration of Hg in a municipal waste landfill leachate can range from 0.05 to 160 μg/l [12,13,14]. Tang et al. [15] proved that sewage treatment system can significantly reduce particulate Hg in leachate, ground water and surface units and evaluated the THg concentration at 0.1–1.02 g/L.

Despite numerous reports devoted to municipal waste management and the resulting hazards, the actual influence of such objects on the basic elements of the environment (water, soil, plants) is still not fully known [6], [16]. Hg properties, such as toxicity, mobility and ability to migrate over large distances, require continuous monitoring of the concentration of this metal, particularly in the vicinity of locations commonly considered as potential sources of pollution by Hg (e.g., municipal waste landfills).

The current study focuses on the influence of a municipal waste landfill on the pollution of basic elements of the natural environment, i.e., soil, water and plants, by Hg. The working hypothesis is that Hg concentration in particular components of the environment increase according to the groundwater flow.

## Materials and Methods

No specific permissions were required for the study area. We obtained permissions to collect samples from waste landfill "Łubna". The field studies did not involve endangered or protected species. Specific location of study: 52°01′49″N 21°08′56″E'

### Study area

**Fig 1:**
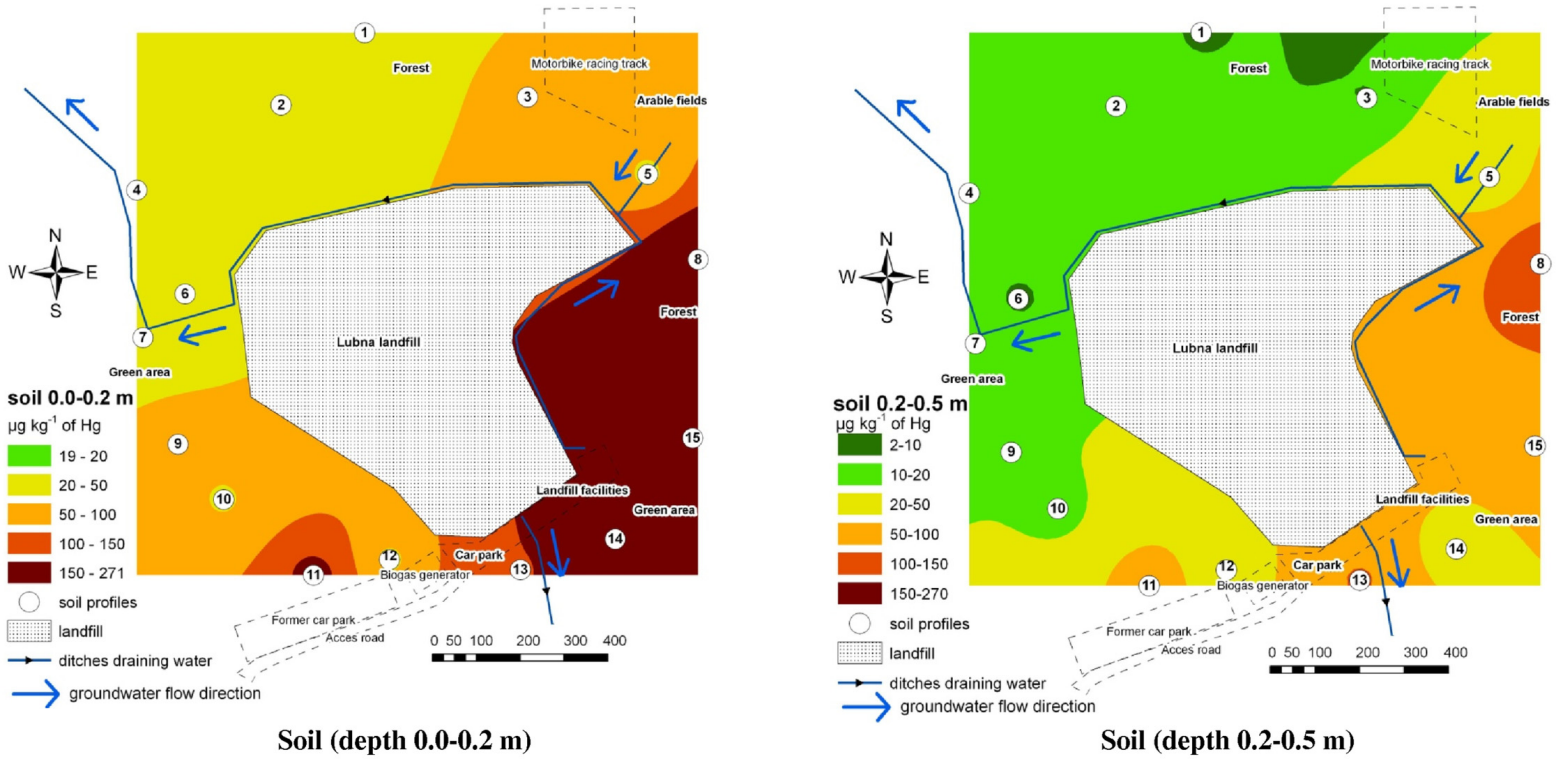
Contamination of the environment with THg based on the accumulated concentration of this element in soil.

**Fig 2:**
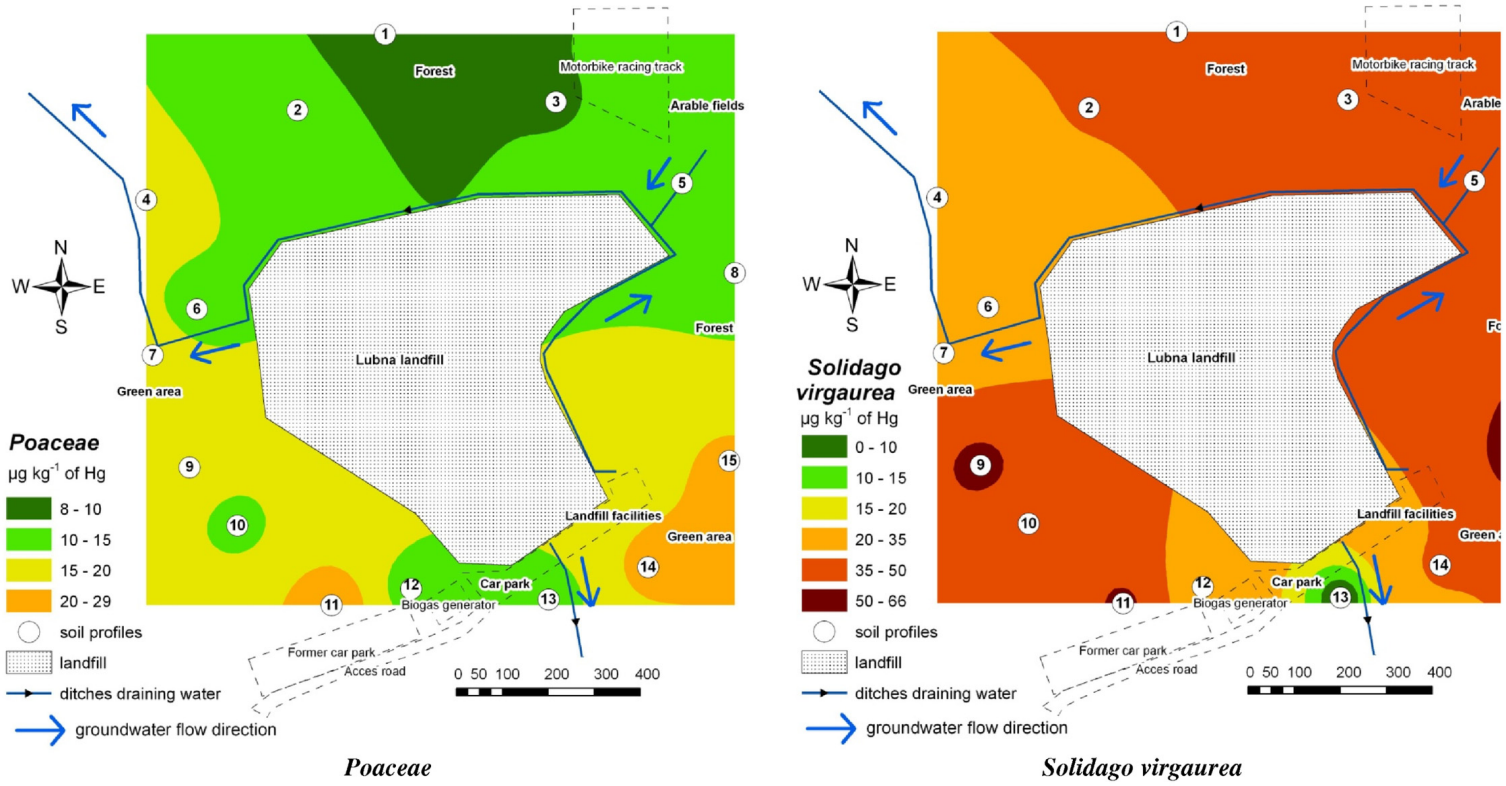
Contamination of the environment with THg based on the accumulated concentration of this element in plants (*Poaceae* sp. and *Solidago virgaurea*).

**Fig 3:**
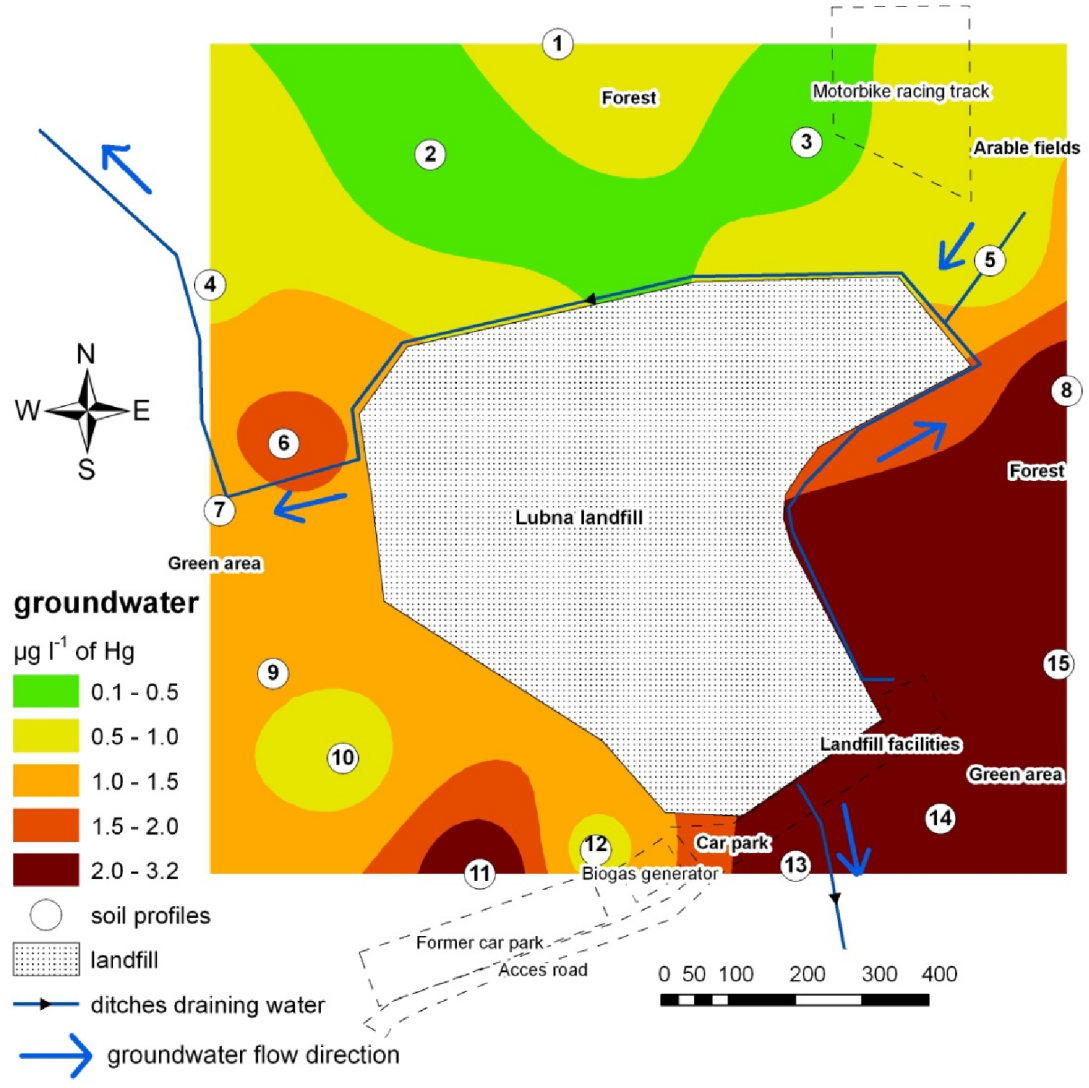
Contamination of the environment with THg based on the concentration of this element in groundwater.

The landfill is located within the watershed on the Warsaw Plain, which is part of the plateau where the Vistula River flood plain is present. The direction of inflow and outflow groundwaters is presented in Figs 1–3. Two major geomorphological formations have been distinguished: post-glacial denudated and non-denudated plateau and river valleys.

Waste disposal on the landfill began in 1978 on an unprepared and moist ground. The first remedial and preserving works were introduced in 1996. They were based on a construction plan and verified and updated according to the observational assessment of the environmental processes occurring on the landfill. During recent years, the daily amount of mixed municipal waste disposed was 400–700 tons, and the peak value achieved was 2500 t/day.

Improvement of groundwater quality was also achieved by introducing leachate transport to waste treatment plants since 1997. In order to treat the waste directly in the locality, a biological treatment plant was introduced at the landfill site [17]. The recognized geological structure of the landfill area shows that the soil is stratigraphically and lithologicallly diverse, i.e. due to its formation, diverse.

The first groundwater table is located within topsoil composed of fluvioglacial sands. The drilled depth of the groundwater table is in the range of 0.1–1.8 m. Groundwater flow is determined by infiltration of rainfall and local drainage. The analyzed aquifer is contaminated by the landfill leachate and contaminants washed out from precipitation, all coming from infiltration through the waste disposed in the landfill from 1978 to 1998 (before construction of a cut-off wall). After the completion of the cut-off wall construction in June 1998, the process of leachate infiltration through the first aquifer and to ditches was successfully eliminated. The existing drainage, network of ditches and cut-off wall bypassing the landfill resulted in significant changes of the groundwater flow direction and velocity, in comparison to the primary pattern of hydroisohypses [18].

Reclamation works on the landfill in the late 1990s included construction of a bentonite cut-off wall sealing the sides of the object, mainly focused on preventing the migration of pollutants in the first aquifer horizon. The scheme of the introduced reclamation solutions is presented in S1 Fig.

### Sampling and methodology

The study material included soil, plants and groundwater samples collected from the landfill vicinity. Fifteen study plots were selected for the study. The area was characterized by a variable morphology: forest areas, grassy wastelands, arable fields, and ditches draining the landfill.

Soil samples were collected from three depth levels from each plot (0–0.2, 0.2–0.5 and 0.5–0.8 m). Twelve samples of soil and plant leaves were collected from each location: grass from the family *Poaceae* sp. and *Solidago virgaurea*. Groundwater was collected using a submersible pump in piezometers used in the monitoring system from depths of 0.25–0.75 m. 15 research areas of 100 m^2^ each, i.e. around each of the installed piezometers, were chosen for this study. Consequently, 12 plots of 1 m^2^ were selected randomly within every research area. 6 soil samples of 0.5 kg each from every analysed depth were collected from all 1 m^2^ plots whereas plant sample was collected from the whole surface of 1 m^2^ plot, i.e. all the plants growing on the plot were cut and analysed. Water samples of 0.5 l each from piezometers were collected 6 times from each analysed depth at the same time, stored in the laboratory refrigerator and analysed on the following day. Mixed sample for analysis was prepared from all the collected individual samples of soil and plant leaves. The material for analysis was dried at 20°C. The obtained results were converted to dry weight determined at 105°C.

The total Hg (THg) content was determined in the plants, soils and groundwater. In the analyzed samples, the THg was determined in an air-dried mass of soil and plants using an AMA-254 Analyzer, which allowed for fast analysis of Hg without the need of an initial stage of preparation. The material for analysis was dried at 20°C. Analytical material was not dried at a higher temperature due to potential loss of mercury during this process. The obtained results were converted to dry weight determined at 105°C, The results of THg in soil and plants were calculated to dry mass.

To provide quality control (QC), the elemental content in the plant samples was determined using certified reference materials from NIST- USA. The obtained results were in good agreement with the certified values. The recovery range was from 97 to 99% and accuracy 2–3%.

Examining biological factors is beneficial to assess the degree of pollution by metals and their accumulation, mobility, translocation, and interaction in the environment. Several parameters were calculated in order to quantitatively characterize the origin and transfer of Hg:

1. Biological Accumulation Coefficient (BAC), which expresses the ratio of metal concentration in plants to its concentration in soil (0–0.2 m);
  $$BAC=\frac{Hg_{plant}}{Hg_{soil}}$$
2. Enrichment Factor (EF_soil_), which is the relative abundance of a chemical element in soil compared to the relative abundance with respect to the local background (50 μg kg^-1^);
  $$EF_{soil}=\frac{Hg_{soil}}{Hg_{soil,\;background}}$$

1. Mobility ratio, which expresses the ratio of metal concentration in soil (0–0.2 m) to its concentration in groundwater;
  $$MR=\frac{Hg_{soil(0-0.2m)}}{Hg_{groundwater}}$$
2. Enrichment Factor in the soil profile.
  $$EF_{prof}=\frac{Hg_{soil(0-0.2m)}}{Hg_{soil(0.2-0.5m)}}$$

### Data analysis

Basic descriptive statistics were used to determine the variability of the studied properties. Additionally, analysis of correlation was conducted, with application of the Spearman rank correlation to determine the relationships between the Hg content in soil, groundwater and plants. Cluster analysis was conducted to group similar variables i.e. Hg concentrations. Euclidean squared distance was used as a measure of multivariate similarity and Ward’s methods was used for agglomeration of objects. Statistical analysis was conducted using the software Statistica 10, with a significance level of 0.05. Maps presenting the Hg content in the investigated area were made by interpolation using Inverse Distance Weighting tools in Geostatistical Analyst of ArcGIS 9.3 software (ESRI, Redlands, CA.).

## Results

The average concentration of THg near the Łubna municipal landfill in the topsoil (0–0.2 m) ranged from 19 μg kg^-1^ d.m to 271 μg kg^-1^ with a median of 52 μg kg^-1^. At the depth of 0.2–0.5 m, the concentration ranged from 3–169 μg kg^-1^ (median at 12 μg kg^-1^), and the concentration was 1–26 μg kg^-1^ (median at 4 μg kg^-1^) in the lowest analyzed horizon (0.5–0.8 m). The THg content in the studied soils decreased with the depth of the soil profile, a fact commonly confirmed in the literature [19,20]. The value of the EF_prof_ in the soil profile (profile 0–0.2 m/0.2–0.5 m) was always higher than 1 (1.2–17.0) on all surfaces.

The concentration of THg in the lowermost layer of the soil (0.5–0.8 m) was much lower than the background value. Therefore, for this layer, a map was not drawn, and the statistical analysis comparing it with other results was not conducted. The distribution of isolines on the maps (Fig 1) indicates that soil in both profiles to the west and south of the landfill contained more THg than in the northern and eastern part of the study area. Land morphology (forest areas, grassy wastelands, arable fields, ditches draining the landfill and surface runoff direction) did not influence the degree of pollution, which indicates that the pollution level was influenced by the deposition of Hg compounds from the air and from earlier years.

The content of THg in plants was less variable than in the soils and reached lower values. Leaves of *Solidago virgaurea* (dicotyledonous plant) contained from 19 μg kg^-1^ to 66 μg kg^-1^ (median at 37.3 μg kg^-1^), and the *Poaceae* sp. (monocotyledonous plants) contained much less, at 8–29 μg kg^-1^ (median at 15.2 μg kg^-1^). The maps (Fig 2) show the THg content in plants from particular surfaces; the isolines have a different distribution. The highest THg concentration was noted in leaves of *Solidago virgaurea*, growing in the north-western part of the study area, and in *Poaceae* sp. from the south-western and south-eastern part of the area. Land management did not influence the THg content in plants. There was no correlation between the THg content in both plant groups (Table 1).

**Table 1:** Correlation coefficients of Spearman correlation between the content of THg in soil, plants and groundwater.

	Soil (depth)		Plant	
	0.0–0.2 m	(0.2–0.5 m)	S. *virgaurea*	*Poaceae* sp.
soil (0.2–0.5 m)	**0.81**			
*S*. *virgaurea*	0.45	0.32		
*Poaceae* sp.	0.52	0.42	0.42	
Groundwater	**0.64**	**0.59**	0.17	0.51

In bold are coefficients indicating statistically significant relationships at significance level α = 0.05.

The Biological Coefficient Accumulation (BAC) values for *Solidago altissima* and topsoil were low, averaging approximately 0.81, exceeding 1 in only two locations. *Poaceae* sp. had an average of 0.30 and did not exceed 1 in any location (Table 2).

**Table 2:** Values of biological factors.

No	BAC^1^	BAC^2^	EF_soil_	EF_prof_	MR
1	2.05	0.42	0.21	2.38	24.1
2	0.69	0.21	0.58	2.48	47.3
3	0.67	0.14	0.70	7.00	175.0
4	0.86	0.53	0.40	3.27	40.0
5	2.22	0.65	0.26	7.67	34.3
6	0.70	0.37	0.30	3.86	14.8
7	0.79	0.62	0.38	1.79	27.4
8	0.15	0.04	3.01	1.60	112.4
9	0.96	0.26	0.77	3.29	70.4
10	0.79	0.32	0.38	17.00	49.3
11	0.29	0.14	2.29	2.22	75.2
12	1.16	0.53	0.21	6.33	27.5
13	0.27	0.08	1.62	1.22	66.7
14	0.18	0.10	2.50	18.75	75.0
15	0.29	0.11	2.20	2.68	62.5

BAC^1^ (Biological Accumulation Coefficient) the ratio of THg concentration in *S*. *virgaurea* to its concentration in soil (0–0.2 m); BAC^2^ (Biological Accumulation Coefficient) the ratio of THg concentration in *Poaceae* sp. to its concentration in soil (0–0.2 m); EF_soil_ (Enrichment Factor), relative abundance of a chemical element in a soil compared to the relative abundance respect to local background (50 μg kg^-1^); EF_prof_ (Enrichment Factor) in soil profile Hg _soil (0–0.2 m)_/Hg _soil(0.2–0.5 m);_ MF (Mobility ratio) expresses the ratio of metal concentration in soil (0–0.2 m),to its concentration groundwater

The content of THg in groundwater was variable, between 0.36 μg l^-1^ and 3.01 μg l^-1^ (median at 0.98 μg l^-1^). The highest pollution was noted in the south-eastern region and the lowest in the northern part of the study area (Fig 3). Land morphology had no influence on the content of THg in groundwater. Statistical analysis of the results has shown a significant relationship between THg content in the soil layers at 0–0.20 m and 0.20–0.5 m and its concentration in groundwater. The ratio between the topsoil and groundwater content (Mobility Ratio—MR) ranged from 24 to 175.

## Discussion

In the literature, the level of Hg geochemical background in the topsoil has a wide range of 20–300 μg kg^-1^ [21]. According to the Forum of European Geological Surveys and the Geochemical Atlas of Europe, the average concentrations of Hg in European soils are 61 μg kg^-1^ (5–1350 μg kg^-1^) [22]. In USA, values of 35 μg kg^-1^, 10–550 μg kg^-1^, and 35 μg kg^-1^ are reported for agricultural land, grasslands, and mixed forests, respectively [23]. The background Hg value of soil in China was shown to be 65 μg kg^-1^ [24], compared to Australia, where it ranged from 1 μg kg^-1^to 100 μg kg^-1^ [25]. In Poland, the background level was assumed to be 50 μg kg^-1^ [26]. The degree of soil pollution in the Łubna landfill area was not high. In the surface soil layer (0–0.2 m), the level of THg pollution on 50% of the study plots was lower than the background for Poland at 50 μg kg^-1^.

In polluted areas, the THg content in soil may be significantly higher. The agricultural soil in Europe contains a maximum of 1150 μg kg^-1^ (median at 0.30 μg kg^-1^), and the grazing land soil maximum value is 3120 μg kg^-1^ (median at 0.35 μg kg^-1^) [27]. The THg content in the topsoil in Beijing varied between 10–966 μg kg^-1^ (median at 185 μg kg^-1^) [28]. In Berlin, the maximum value was 7120 μg kg^-1^ (median at 190 μg kg^-1^), while the soil samples taken in the surroundings of Berlin had a median value of 50 μg kg^-1^ THg [29]. THg concentrations and distribution in soil around Hg mines in the Big Bend region, Texas (USA), approximately 300 m from an inactive THg mine, contained elevated concentrations at 3800–11000 μg kg^-1^, which were considerably higher than the THg in soil collected from the baseline sites at 30–50 μg kg^-1^ located 24 km from the mines [30]. The level of soil contamination by THg in the Łubna MSW area may be defined as low.

In Poland, the THg content in soils was also variable. The Geochemical Atlas of Poland [31] reported soil values from < 50 μg kg^-1^ to 7550 μg kg^-^1 and arable soil values from < 50 μg kg^-1^ to 4750 μg kg^-1^. In Gdańsk, the THg concentrations in the soil of the burial ground ranged from 37 to 4817 μg kg^-1^ [32]. Arable soil in an industrialized area (Upper Silesia) contained 20–460 μg kg^-1^ of THg (median at 60 μg kg^-1^) [33] and in the Legnica-Głogów copper basin, soils from industrialized areas contained a maximum of 5130 μg kg^-1^, while forest soils contained 1970 μg kg^-1^ and soils from arable lands had 2740 μg kg^-1^ [34].

In the EU directive [35], a maximum tolerable THg content in fodder such as grass is 100 μg kg^-1^. The THg content in the studied soils was much lower in all samples than the value admissible by the EU directive, with a maximum of 66 μg kg^-1^. De Temmerman et al. [36] determined the background concentration in *Lolium perenne* at 5–20 μg kg^-1^, and Carpi et al. [37] noted higher background concentrations of 33 μg kg^-1^ in *Lolium multiflorum*.

Plants accumulate Hg mainly from air deposition [38,39,40]. Tomiyasu et al. [41] did not find any relationship between the THg content in leaves of *Solidago altissima* and its concentration in soil. Similarly, Niu et al. [42] noted a lack of relationship for *Lolium perenne*, who suggested the application of this plant in biomonitoring of Hg pollution in air

The background level of THg in groundwater was < 1.00 μg l^-1^ [43,44]. The concentration of THg in groundwater, depending on the location, attained different values. For example, in Poznań (Poland), it reached 0.8–4.1 μg l^-1^, with a mean of 1.3 μg l^-1^ [45]. In Bavaria, 3 MSW landfills showed values <0.04 μg l^-1^ [46]. In New Jersey (USA), in an urbanized area, the concentration ranged from 0.3–1980 0.04 μg l^-1^ [47]. In the Kathmandu Valley (Nepal), in an industrial area, the maximum value was 300 μg l^-1^ [48].

The total mercury concentration in leachates from MSW landfills is also variable. Matwiejczyk et al. [49] determined concentrations of THg at 0.3–0.7 μg l^-1^ in leachates from 22 MSW landfills in Upper Silesia (Poland), while Kulikowska & Klimiuk [13] noted concentrations of 17 μg l^-1^ in MSW landfills in Bartoszyce (Poland). Øygard et al. [50] found concentrations of 0.013–0.027 μg l^-1^ from four MSW landfills in Norway, Ilgen et al. [46] noted < 0.04–1.9 μg l^-1^ in 12 MSW landfills in Bavaria (Germany), Olivero-Verbel et al. [51] noted <0.015 μg l^-1^ in Cartagena town (Colombia), The maximal admissible concentration of THg in potable water in EU countries [52] is 1 μg l^-1^, whereas Polish Law [53] allows an annual mean concentration of THg at 0.05 μg l^-1^ in surface waters. The hydrogeochemical background for THg in groundwater in Poland is 0.05–1.00 μg l^-1^, whereas the range of 1.0–5.0 μg l^-1^ was determined for water of insufficient quality due to natural processes and human activity.

**Fig 4:**
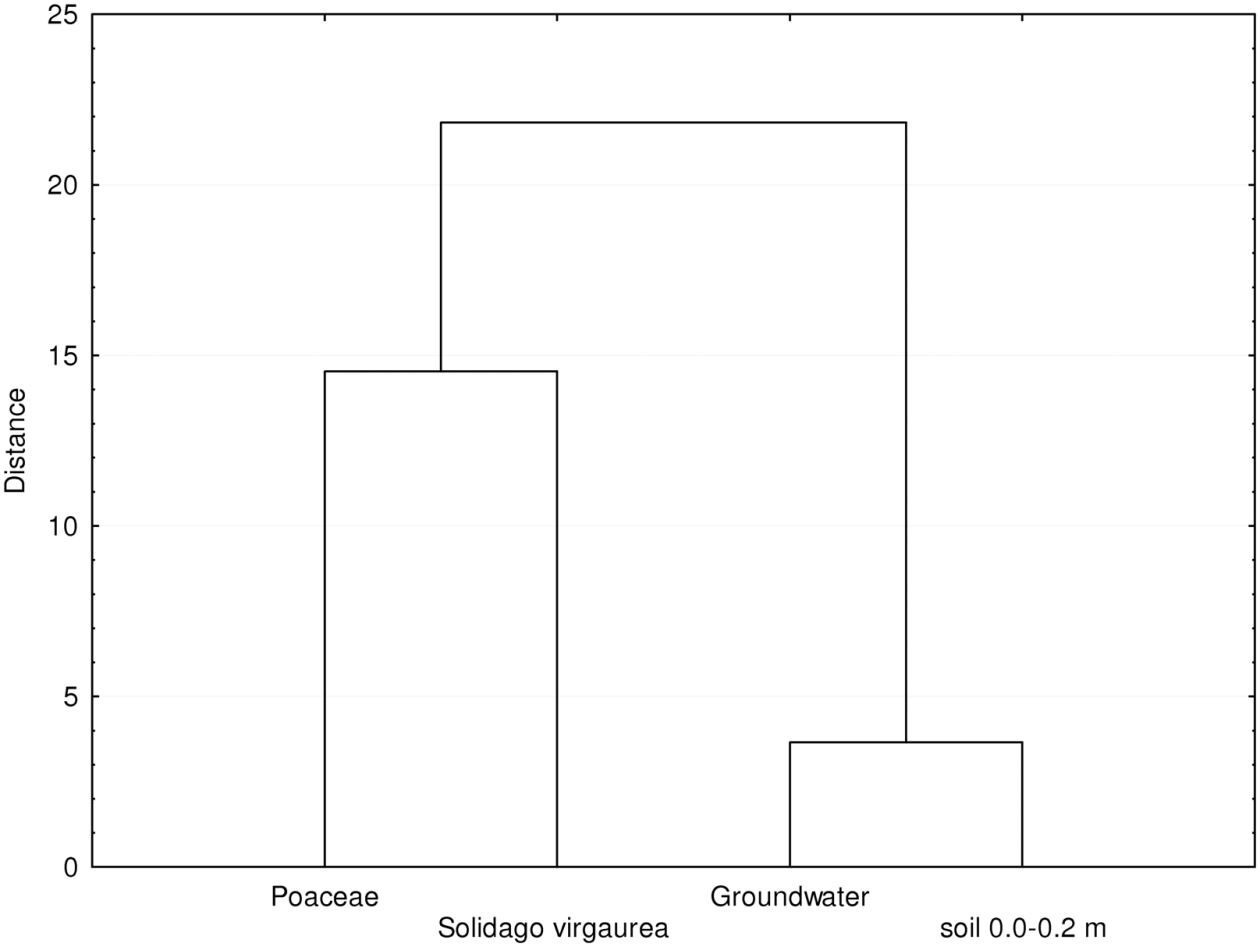
Results of cluster analysis determining the similarities between the THg content in various objects.

Groundwater is considered a source of Hg distribution from MSW landfills [13,47, 49]. The obtained results indicate that the groundwater pollution near the MSW landfill is higher than the background level. However, it is not high, and only 50% of the samples exceed the admissible level for potable water. The isoline distribution on the map does not indicate unambiguously that the landfill is the source of pollution. The statistical analysis of the results points to a significant relationship between the THg content in soil layers of 0–0.2 m and 0.2–0.5 m and its concentration in groundwater. Based on the dendrogram (Fig 4), the THg content in groundwater is correlated to the topsoil content, whereas it is not related to the THg content in plants. The content in *Poaceae* sp. and *Solidago virgaurea* was rather poorly related, which is indicated by the level at which the dendrogram branches are connected. The research showed that the construction of cut-off barrier bypassing MSW landfill decreased emission of Hg into the waters outflowing from the landfill. This has been proven by low content of Hg in the soil and water as well as in plants.

## Conclusions

Environmental pollution near the Łubna MSW landfill reflected in the content of THg in three soil layers, plants and groundwater was relatively low. The concentration of Hg in soil decreased with depth, and in the lowest analyzed layer (0.5–0.8 m), it was below the background level. In the topsoil (0–0.2 m), the level of pollution by THg on 50% of the analyzed surfaces was lower than the background level of 1.0 μg l^-1^ and did not exceed the admissible level for potable water in Poland (1 μg l^-1^).

Plants accumulate Hg mainly from air deposition. The statistical analysis indicated a lack of connection between THg content in soil and plants (*Solidago virgaurea* and *Poaceae* sp.). Leaves of the dicotyledonous plant *Solidago virgaurea* contained more THg (median at 37.3 μg kg^-1^) than the monocotyledonous *Poaceae* sp. (median at 15.2 μg kg^-1^). The content of THg in both plant groups was much lower in all samples than the maximum, tolerable Hg content in fodder given in a EU directive at 100 μg did does not indicate the influence of the land morphology (i.e., forest areas, grassy wastelands, arable fields, direction of runoff surface water and ditches draining the landfill) to THg content in soil, plants and groundwater. It also did not point to the landfill as a direct pollution source. Deposition of Hg compounds from the atmosphere and the influence of other historical pollution sources controlled the pollution level in specific locations.

## Supporting Information

S1 Fig Scheme of the reclamation belt surrounding the landfill. (TIF)
