# Supplementary figures and images for: Influence of a Municipal Waste Landfill on the Spatial Distribution of Mercury in the Environment
Source: PLoS One. 2015 Jul 15;10(7):e0133130. doi: 10.1371/journal.pone.0133130 (PMC4503673; doi:10.1371/journal.pone.0133130)

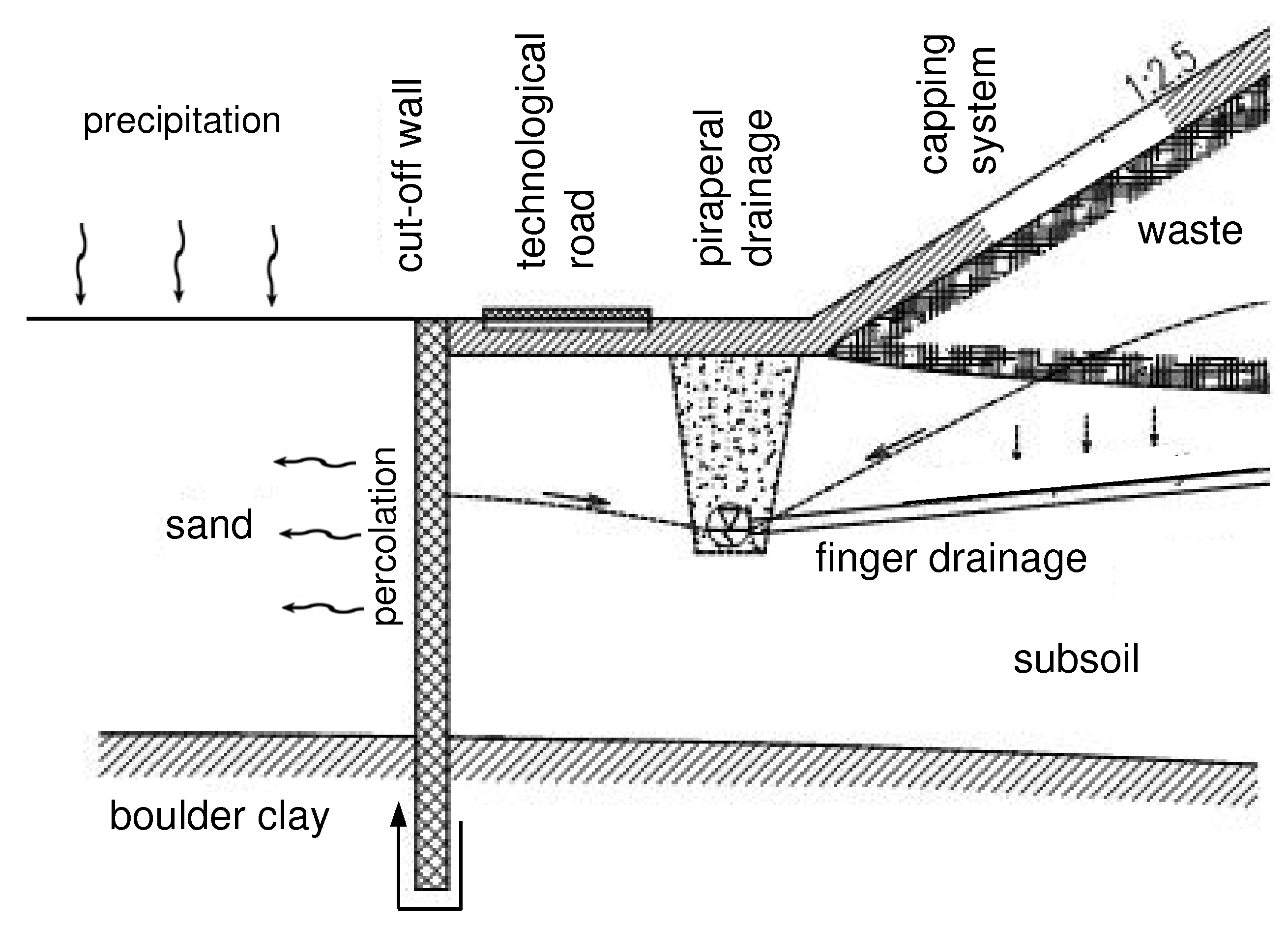

Supplement: S1 Fig — (TIF) [file pone.0133130.s001.tif]
